# Psychometric Properties of an Arabic Pain Anxiety Symptoms Scale-20 (PASS-20) in Healthy Volunteers and Patients Attending a Physiotherapy Clinic

**DOI:** 10.1007/s12529-016-9608-1

**Published:** 2016-11-10

**Authors:** Osama A. Tashani, Oras A. AlAbas, Raafat A.M. Kabil, Mark I. Johnson

**Affiliations:** 1 Centre for Pain Research, Leeds Beckett University, Leeds, UK; 2 MENA research group, Leeds Beckett University, Leeds, UK; 3 Sirte University, Sirte, Libya; 4 Sohag University, Sohag, Egypt

**Keywords:** Pain, Anxiety, Fear of pain, Psychometric analysis, Libya

## Abstract

**Purpose:**

The aim of this study was to cross-culturally adapt the PASS-20 questionnaire for use in Libya.

**Methods:**

Participants were 71 patients (42 women) attending the physiotherapy clinic, Ibn Sina Hospital, Sirt, Libya for management of persistent pain and 137 healthy unpaid undergraduate students (52 women) from the University of Sirt, Libya. The English PASS-20 was translated into Arabic. Patients completed the Arabic PASS-20 and the Arabic Pain Rating Scales on two occasions separated by a 14-day interval. Healthy participants completed the Arabic PASS-20 on one occasion.

**Results:**

The internal consistency (ICC) for pain patient and healthy participant samples yielded a good reliability for the total score, cognitive anxiety, fear of pain, and physiological anxiety. The test-retest reliability of the Arabic PASS-20 score showed high reliability for the total score (ICC = 0.93, *p* < 0.001), escape/avoidance (ICC = 0.93, *p* < 0.001), fear of pain (ICC = 0.94, *p* < 0.001), and physiological anxiety subscales (ICC = 0.96, *p* < 0.001) and good reliability for the cognitive anxiety (ICC = 0.85, *p* < 0.001). Inspection of the Promax rotation showed that each factor comprised of five items were consistent with the theoretical constructs of the original PASS-20 subscales.

**Conclusion:**

The Arabic PASS-20 retained internal consistency and reliability with the original English version and can be used to measure pain anxiety symptoms in both pain and healthy individual samples in Libya.

## Introduction

Psychosocial factors such as fear of pain, catastrophizing, depression, and anxiety are determinants of differences in pain responses. Anxiety, a negative emotional response to an anticipated threat, is linked with increased pain sensitivity in patients with chronic pain [1] and in pain-free individuals exposed to painful stimuli [2]. Studies on patients with musculoskeletal pain have found that aspects of behaviour such as fear-avoidance beliefs, pain-related fear, and thought suppression are associated with pain and disability [1]. Indeed, fear of pain and fear-avoidance behaviour were developed as a concept to explain exaggerated pain perceptions in patients and might help in understanding how and why some individuals with musculoskeletal pain develop a chronic pain syndrome [3]. Anxiety that is specifically relevant to pain, called pain-related anxiety, is more likely to be correlated with pain sensitivity than general anxiety [4].

Several measures are used to assess pain-related anxiety. The multi-dimensional Pain Anxiety Symptom Scale (PASS) is one of the most commonly used pain-specific anxiety scales. The PASS is a 40-item self-report scale which was developed to measure the fear of pain along four dimensions; fearful interpretations, avoidance and escape, physiological responses, and cognitive interference [5, 6]. There is strong empirical evidence that the PASS is associated with pain severity and other measures such as patient functioning [4]. In addition, several psychometric studies have shown that the four dimensions (factors) of the PASS are consistently reproducible in patients and healthy participants [6]. McCracken et al. found that scores from the PASS and the Fear Avoidance Beliefs Questionnaire (FABQ) “accounted for more variability in pain, disability, and pain behaviour compared with scores from the FPQ [Fear of Pain Questionnaire] and STAI [the State-Trait Anxiety inventory]” [6]. This suggests that the PASS is a more appropriate tool to measure multiple anxiety response categories toward pain and is preferable than measuring general response tendencies.

The short form of the PASS is a 20-item self-report scale that measures four components of fear of pain, including cognitive anxiety, escape and avoidance, fearful appraisals of pain, and physiological anxiety (PASS-20, Appendix 1, (8)). Items are rated for frequency of occurrence on a 6-point Likert scale anchored at 0 (never) and 5 (always), providing scores for the four dimensions and total. The PASS-20 has been validated in chronic pain patients [7] and healthy individuals [8]. Cross-cultural adaptation of the questionnaire to other languages has been performed in South Korea [9], Germany [10], and China [11]. To our knowledge, there are no published reports of the linguistic validity of the PASS-20 when administered to Arabic populations. There is a need to develop an Arabic version of the PASS-20 to facilitate multinational studies and to compare research results between countries. A protocol of forward and back translation, cultural adaptation, and scale validation is required [12].

The aim of this study was to cross-culturally adapt the PASS-20 questionnaire for use in Libya. The objectives of this study were to 1) cross-culturally adapt the PASS-20 questionnaire for use in Arabic-speaking populations, 2) test the psychometric properties of the Arabic PASS-20 with the original English PASS-20, and 3) compare Arabic PASS-20 responses of pain patients and healthy pain-free participants.

## Methods

The study was approved by the medical research ethics committee at the University of Sirt, Libya and the research ethics committee at Leeds Beckett University, UK. The protocol for translating the PASS-20 followed the guidelines for cross-cultural adaptation of self-report measures [12]. The analysis of reliability and validity was conducted on the final Arabic version of the PASS-20.

### Phase 1: Translation and Cross-Cultural Adaptation

1. Translation: The PASS-20 was translated from English to Arabic independently by two bilingual academics who were fluent to a professional level in English language and whose mother tongue was Arabic. Permission to undertake the translation was given by the author of the PASS-20; Lance McCracken, King’s College, London, United Kingdom.
2. Synthesis of the translation: The two translations of the PASS-20 were synthesized to develop a consensus version.
3. Back translation: The re-conciliated Arabic version was back translated into the source language by a professional Arabic-English translator who was blind to the original version.
4. Expert committee review: A psychologist and two academics not involved in the translation reviewed all translated versions of the PASS-20 and discussed possible discrepancies. They developed the final version of the Arabic PASS-20 (Appendix 2).
5. Pre-testing: The translated questionnaire was pretested on 60 healthy individuals who participated in a cold pressor pain experiment previously described by Tashani et al. [13].

### Phase 2: Psychometric Testing Including Reliability and Validity of the Arabic PASS-20

#### Enrolment of Patients and Healthy Participants

Seventy-one patients (42 women, mean age (SD) = 32 [10] years) with persistent pain who had been referred by their physicians to the physiotherapy clinic at Ibn Sina Teaching Hospital, Sirt, Libya took part in the study. Medical notes were used to confirm that all patients had recurring episodes of pain of more than 6 months before referral. Twenty-four of these patients (34%) were diagnosed as having low back pain, 13 (19%) had postoperative or traumatic pain comprised, and 9 (13%) had osteoarthritis or rheumatoid arthritis. The rest of the patients had neck pain, shoulder pain, lower leg pain, and foot pain. Only three patients had multiple sources of pain.

One hundred and thirty-seven healthy pain-free unpaid undergraduate students from the University of Sirt, Sirt, Libya (52 women, mean age (SD) = 21 [2] years) were recruited via announcements in lectures and noticeboard advertisements. The healthy participants served as a control group to compare the PASS-20 scores with pain patients and to test the discriminatory power of the instrument within the two sample populations. To be eligible for the study, healthy participants had to have no previous chronic pain complaints.

To test-retest the reliability of the Arabic PASS-20, patients completed the Arabic PASS-20 and an Arabic Pain Rating Scale (PRS, available from the British Pain Society website) on two occasions separated by a 14-day interval. Patients received physiotherapy treatment that was tailored to their needs during this interval. Healthy participants completed the Arabic PASS-20 on one occasion. Participants in both groups were instructed to report any words that they found to be unclear. Data for the study was collected before the conflict in Libya.

### Measurements

#### Background Variables

Patients and healthy participants completed a questionnaire to document age, sex, marital status, education, financial status, and course of pain.

#### Pain Rating Scales

Patients completed pain rating scales to assess 1) present pain intensity, 2) pain intensity in the last week, 3) distress due to present pain, 4) distress due to pain during last week, and 5) pain interference with daily life. Each scale was scored between 0 (no pain intensity, distress or interference) and 10 (the worst possible outcome). In addition, patients answered the question “If you have had treatment for your pain, how much has this relieved (taken away) the pain?” where 0% represented no relief of pain and 100% represented complete relief of pain.

#### Pass-20

The 20-item PASS-20 has four subscales measuring factorial distinct components of pain-related anxiety. The cognitive subscale assesses cognitive anxiety symptoms, such as racing thoughts and impaired concentration due to pain; the fear subscale assesses fearful thoughts and anticipated negative consequences of pain; the escape/avoidance subscale assesses escape and avoidance of actions that may cause pain; and the physiological anxiety subscale assesses physiological arousal in response to pain [7]. Respondents rate each item on a six-point scale ranging from 0 (never) to 5 (always). Total scores range from 0 representing no pain anxiety to 100 representing severe pain anxiety.

### Data Analysis

Factor analysis was conducted to determine whether the Arabic version of the PASS-20 had the same number of factors as the English version. It has been recommended that the sample size to undertake such an analysis should be approximately five times the number of items in the instrument [14]. Mean, standard deviation, and 95% confidence interval were used to express the variability of data, and the Kolmogorov-Smirnov was test used to check whether data was normally distributed.

The internal consistency of items in the Arabic PASS-20 was assessed using the Cronbach’s alpha, which indicates the extent to which a set of test items can be treated as measuring a single latent variable. Item-to-total correlations of the Arabic PASS-20 were assessed using the Spearman correlation coefficients. Intra-Class Correlation Coefficients (ICC) were used to test-retest reliability according to the method described by Bland and Altman [15, 16]. Differences in scores of the Arabic PASS-20 scores taken on two occasions for patients were analysed using the paired sample *t* test.

Exploratory factor analysis was carried out to explore the dimensionality of the Arabic PASS-20. The Kaiser-Meyer-Olkin and the Barlett test of sphericity were performed to determine the sampling adequacy for principle components analysis (PCA) [14]. A PCA was then performed to determine if the four dimensions (subscales) could represent four distinct variables. Oblique (Promax) rotation was applied to minimize the complexity of loading for each component. For each item, acceptable construct validity was defined as loading of 0.3 or greater on the first principle component with a 0.10 or greater difference in loadings with the other factors. The scree test and the eigenvalues (above 1) were used to identify the number of factors. The factor model was then tested in the two groups (pain patients and healthy pain-free participants) using a confirmatory factor analysis (CFA). A CFA with maximum likelihood was conducted to confirm that the measurement properties of the original version of the instrument applied to the Arabic version and to compare the model obtained with the original factor model of the PASS-20. The fit of the CFA model was assessed using the chi-squared test where ×^2^/df values should be less than 3.0; the Comparative Fit Index (CFI) where values close to or greater than 0.90 reflect a good fit to the data; the Root-Mean-Square Error of Approximation (RMSEA) where values of less than 0.05 indicate a good fit and values as high as 0.08 represent reasonable errors of approximation; and the Expected Cross Validation Index (ECVI) to compare the competing models with lower values indicating better fit [10].

For pain patients, a series of intraclass correlations were calculated to examine the correlation between pain rating scales of the first and second assessments and to examine the effect sizes of the correlations between the Arabic PASS-20 total score, subscales, and pain severity. Cohen’s guidelines were used to categorize the strength of the correlation coefficient [8]. An independent *t* test analysis was applied to the Arabic PASS-20 total score and subscale scores to determine if there were differences between pain patient and healthy pain-free participants. Significance level was set at *p* < 0.05 (two-tailed). Data analyses were performed using the SPSS and AMOS Version 19.0 (IBM, Ottawa, Canada).

## Results

### Phase 1: Translation and Cross-Cultural Adaptation

Verbal feedback from pain patients and healthy pain-free participants suggested that the Arabic PASS-20 questionnaire was easy to understand, and they reported no difficulty in completing it.

### Phase 2: Psychometric Testing Including Reliability and Validity

The internal consistency for the pain patient and healthy pain-free samples yielded good reliability for the total score and for the subscales cognitive anxiety, fear of pain, and physiological anxiety (Table 1). Low internal consistency was found for the subscale escape/avoidance in both samples (0.60 and 0.65, respectively). The ICC of the instrument was further supported by the item-to-total correlation which fell within the desirable range (0.2 to 0.8). Item 6, “I try to avoid activities that cause pain,” was poorly correlated with the total of the remaining items (0.13) in the healthy sample. An item-total correlation of 0.60 was found for item 6 in the pain patient sample. The test-retest reliability showed high reliability and significance for the total score (ICC = 0.93, *p* < 0.001) and for the subscales scape/voidance (ICC = 0.93, *p* < 0.001), fear of pain (ICC = 0.94, *p* < 0.001), and physiological anxiety (ICC = 0.96, *p* < 0.001). There was good reliability for the subscale cognitive anxiety (ICC = 0.85, *p* < 0.001). The stability of the instrument was supported by a paired sample *t* test that indicated no significant differences in total and subscale scores over a 14-day period.

**Table 1:** Internal consistency in the two groups Arabic PASS-20 and test-retest reliability of the questionnaire in patient group only

Items	Healthy (*n* = 137)		Pain patients (*n* = 71)		
	Item-to-total correlation	Cronbach’α*	Item-to-total correlation	Cronbach’α*	Intraclass coefficient
Cognitive anxiety		0.71		0.78	0.85
I can’t think straight when in pain	0.45		0.54		
During painful episodes it is difficult for me to think of anything besides the pain	0.53		0.54		
When I hurt I think about pain constantly	0.35		0.65		
I find it hard to concentrate when I hurt	0.40		0.46		
I worry when I am in pain	0.34		0.51		
Escape Avoidance		0.60		0.65	0.93
I go immediately to bed when I feel severe pain	0.13		0.60		
I will stop any activity as soon as I sense pain coming on	0.46		0.46		
As soon as pain comes on I take medication to reduce it	0.20		0.54		
I avoid important activities when I hurt	0.29		0.44		
I try to avoid activities that cause pain	0.31		0.37		
Fear of Pain		0.72		0.81	0.94
I think that if my pain gets too severe, it will never decrease	0.27		0.54		
When I feel pain I am afraid that something terrible will happen	0.40		0.71		
When I feel pain I think that I might be seriously ill	0.40		0.73		
Pain sensations are terrifying	0.21		0.65		
When pain comes on strong I think that I might become paralysed or more disabled	0.41		0.60		
Physiological Anxiety		0.73		0.70	0.96
I begin trembling when engaged in an activity that increases pain	0.40		0.65		
Pain seems to cause my heart to pound or race.	0.35		0.50		
When I sense pain I feel dizzy or faint	0.26		0.38		
Pain makes me nauseous	0.34		0.52		
I find it difficult to calm my body down after periods of pain	0.40		0.63		
Total score		0.84		0.88	0.93

*p<0.001

### Factor Analysis

Table 2 presents the factor solution in the healthy group (*n* = 137). The Kaiser-Meyer-Olkin test (KMO) was found to be 0.75, which exceeds the recommended minimum value of 0.60 (Kaiser 1974). Barlett’s test of sphericity was highly significant (Barlett’s χ^2^ (66) = 730.32, *p* < 0.001) showing that the data was appropriate for the PCA. Items that loaded 0.3 or greater on each factor were retained within that factor. Both the scree test and eigenvalues of 5.11, 1.66, 1.50, and 1.45 indicated a four-factor solution and explained 50.2% of the total variance. The factors were labelled as follows with the explained variance in parentheses: 1 cognitive anxiety (26.60%), 2 fear of pain (7.29%), 3. escape/avoidance (7.72%), and 4. physiological anxiety (8.58%). Inspection of the Promax rotation showed that each factor was comprised of five items which were consistent with the theoretical constructs of the original PASS-20 subscales. Additionally, initial item communalities (h^2^) were moderate, ranging from 0.39 to 0.66 [17] and at least half of the items of each factor had a factor loading of 0.60 or greater, which supported the factor stability of the Arabic PASS-20. However, item 10, “I try to avoid activities that cause pain,” showed [17] h^2^ of 0.17 and a loading of 0.25, but it was not excluded from the final Arabic PASS-20. The results of the CFA for the Arabic PASS-20 are shown in Table 3. The χ^2^/*df*, CFI, IFI, RMSA, and ECVI values indicated a better fit for the healthy group compared with the pain patients.

**Table 2:** Factor loadings of the Arabic version of the PASS-20 questionnaire after oblique (Promax) rotation

Item no.	Item wording	Factor loading				
		Cognitive	Fear	Escape/avoidance	Physiological	h2
1	I can’t think straight when in pain	0.786				0.663
2	During painful episodes it is difficult for me to think of anything besides the pain	0.701				0.705
3	When I hurt I think about pain constantly	0.640				0.428
4	I find it hard to concentrate when I hurt	0.780				0.609
5	I worry when I am in pain	0.445				0.387
6	I go immediately to bed when I feel severe pain		0.487			0.242
7	I will stop any activity as soon as I sense pain coming on		0.635			0.575
8	As soon as pain comes on I take medication to reduce it		0.523			0.440
9	I avoid important activities when I hurt		0.751			0.553
10	I try to avoid activities that cause pain					0.168
11	I think that if my pain gets too severe, it will never decrease			0.713		0.616
12	When I feel pain I am afraid that something terrible will happen			0.776		0.670
13	When I feel pain I think that I might be seriously ill			0.556		0.482
14	Pain sensations are terrifying			0.563		0.419
15	When pain comes on strong I think that I might become paralysed or more disabled			0.605		0.566
16	I begin trembling when engaged in an activity that increases pain				0.617	0.516
17	Pain seems to cause my heart to pound or race.				0.539	0.497
18	When I sense pain I feel dizzy or faint				0.659	0.394
19	Pain makes me nauseous				0.612	0.530
20	I find it difficult to calm my body down after periods of pain				0.583	0.576
Percent of variation		26.597	7.290	7.715	8.579	
Cumulative variance (%)		26.597	33.887	41.602	50.181

**Table 3:** Goodness-of-fit indices for the Arabic PASS-20 factor models

Group	χ2	df	χ2/*df*	RMSEA	CFI	IFI	TLI
Healthy (*n* = 137)	256.79	164	1.57	0.065	0.85	0.85	0.82
Pain patients (*n* = 71)	278.60	164	1.70	0.1	0.77	0.78	0.73
Abrams et al (8) (4-factor model)			1.69	0.07	0.92		

### Group Differences

The Arabic PASS-20 scores were normally distributed for pain patient and healthy pain-free groups (Table 4). Differences between pain patients and healthy participants in the Arabic PASS-20 total and subscale scores were particularly evident for the fear of pain subscale, demonstrating that pain patients reported significantly higher scores than the healthy group (Mean (SD) = 14.76 (7.29) versus 10.94 (6.04), respectively, *p* < 0.001, independent *t* test).

**Table 4:** Means, standard deviations of the Arabic PASS-20 for healthy and patient samples

Subscales	Healthy (*n* = 137)	Pain patients (*n* = 71)	Comparison
	Mean (SD)	Mean (SD)	*p* value
Cognitive anxiety	16.64 (5.74)	17.75 (5.49)	0.183
Escape/avoidance	14.37 (5.55)	15.48 (6.01)	0.187
Fear of pain	10.85 (6.16)	14.77 (7.29)	0.001
Physiological anxiety	11.02 (6.25)	9.59 (6.38)	0.122
PASS-20 total scores	52.94 (17.80)	57.58 (19.80)	0.090

As expected, significant correlations were found between the Arabic PASS-20 total score and pain rating scales (Table 5). Specifically, the Arabic PASS-20 total score positively correlated with present pain intensity (*r* = 0.32, *p* = 0.007), pain intensity in the last week (*r* = 0.27, *p* = 0.024), distress due to present pain (*r* = 0.31, *p* = 0.009), distress due to pain during the last week (*r* = 0.26, *p* = 0.030), and pain interference with daily life (*r* = 0.36, *p* = 0.002). Interestingly, only cognitive anxiety and fear of pain subscales demonstrated significant correlations ranging between medium to large with pain rating scales. No significant correlations were found between the Arabic PASS-20 total score or subscales and pain relief.

**Table 5:** Correlations between pain rating scales (PRS) and the Arabic PASS-20 scores in the patients’ group

Correlations	Cognitive anxiety	Escape/avoidance	Fear of pain	Physiological anxiety	Arabic PASS-20 total score
Present pain intensity	0.39*	0.03	0.34*	0.23	0.32
Pain intensity in the last week	0.34*	−0.02	0.29*	0.22	0.27
Distress due to present pain	0.52*	0.02	0.28*	0.17	0.31*
Distress due to pain during last week	0.49*	0.01	0.21	0.13	0.26*
Pain interference with daily life	0.42*	0.14	0.35*	0.22	0.36*
If you have had treatment, how much has this relieved the pain	0.05	0.16	−0.18	−0.07	−0.03

*p > 0.05

## Discussion

This study found that an Arabic language version of the PASS-20 completed by a sample of Libyan patients with chronic pain performed satisfactorily on all the components of analysis of reliability and linguistic validity. Support for the reliability of the Arabic PASS-20 was based on internal consistency, item-total correlations, and test-retest reliability. Internal consistency was found to be good for the total score and acceptable for the subscales, although the internal consistency of escape/avoidance indicated relatively lower stability of this subscale.

The Cronbach’s alpha of the escape/avoidance subscale was the lowest out of the four dimensions (subscale); for the pain patients and the sample of healthy pain-free university students indicating there is a discrepancy between the five items in this dimension. Low internal consistency in escape/avoidance compared to the total PASS-20 was also found by McCracken and Dhingra [18] (Cronbach’s α 0.75 vs 0.91) and Abrams et al. [8] (Cronbach’s α of 0.67 vs 0.91). Further analysis of our data showed that the escape/avoidance subscale also had the lowest item-total correlations and included the only item that did not meet the criterion for factor loading (item 10, “I try to avoid activities that cause pain”), possibly reflecting cultural differences in interpreting some words. A low internal consistency of the subscale escape/avoidance seems to be a common finding of studies on European participants [10, 19]. The reliability of the scale in the pain patients and healthy pain-free participants was further strengthened through item-total correlation [20]. Test-retest reliability was well established in the pain patient sample with an ICC of 0.93 for the total score and from 0.76 to 0.89 for the subscales. The stability of the Arabic PASS-20 was also confirmed by the paired *t* test that found no significant differences in scores over a 14-day period. This finding indicates that the Arabic PASS-20 has an adequate reliability.

Dimensionality of the Arabic PASS-20 was determined using an exploratory factor analysis (EFA), and this confirmed a four-factor structure consistent with previous studies on in clinical [7, 18] and healthy pain-free [8] populations from Europe and Canada. These four factors replicate the factor structure of the original English PASS-20. The first factor emerging from the present analysis was physiological anxiety, followed by fear of pain, cognitive anxiety, and escape/avoidance. The results of the confirmatory factor analysis (CFA) were supportive of the structures suggested by EFA and comparable to the 4-factor model established in the European and Canadian populations using clinical [7, 19] and healthy samples [8]. Given the above, the Arabic PASS-20 appears to be comparable with the original English PASS-20.

There were no significant differences between men and women in the Arabic PASS-20 total scores or for the subscales cognitive anxiety, escape/avoidance, and fear of pain. Similar results have previously been reported in samples of chronic pain patients [21] and healthy pain-free students [8], although there was a significant difference between men and women in the subscale cognitive anxiety in the study by Keogh et al. [20]. It has been previously reported that women have higher state anxiety and trait anxiety than men [22]. When the Arabic PASS20 was correlated with PRS, stronger associations were found with cognitive anxiety.

Chronic pain patients and healthy pain-free students in our Libyan sample reported higher total and subscale Arabic PASS-20 scores compared with chronic pain patients [18] and healthy pain-free students [8] from Europe, suggesting that Libyans may be more anxious about pain than Europeans. Libyan pain patients had higher subscale scores for fear than the healthy pain-free students, probably because they are not experiencing persistent pain affecting the quality of their life. There is evidence that ethnicity and culture influence pain-related anxiety. Weisenberg et al. [2] found that white Europeans showed a lower level of trait anxiety measured on STAI compared with Puerto Ricans and African Americans. Black patients reported higher levels of pain-related anxiety than White patients, although there were no differences in physical, psychosocial, and total disability. In the UK, Watson et al. [6] claimed that South Asian men reported higher PASS-20 scores than White British counterparts, although there were no statistically significant differences between the groups. The findings from our study suggest that Libyans may be more likely to generate catastrophic thoughts, such as fear of dying or being seriously ill and impaired thinking and concentration, than Europeans. Further studies investigating ethnic variation in anxiety levels are required to confirm this premise.

### Limitations

There were a number of potential shortcomings in this study that might restrict the generalizability of the findings. The sample size of the pain patient group was small. There was a marked difference in the age of participants in the pain patient sample and the healthy pain-free sample. Treatment received by pain patients in the 14-day interval between completing the Arabic PASS-20 was not documented, although there was no correlation to the question “If you have had treatment, how much has this relieved the pain” and the total Arabic PASS-20 score. Thus, any treatment taken within the 14-day interval is unlikely to have had any impact on pain-related anxiety. It was not possible to evaluate the construct validity of the Arabic PASS-20 because we did not administer other instruments that assess pain-related anxiety symptoms. The goodness of fit measures did not represent a good fit possibly because of the small sample size. This was also reflected by the fact that the CFA model showed a better fit for the healthy pain-free participants because of the larger sample size. We recommend that future studies should evaluate the Arabic PASS-20 on large samples of patients with different conditions and that measures of patient function should be included in the study design.

## Conclusion

The Arabic version of the PASS-20 developed in this study retained internal consistency and reliability with the original English PASS-20 and can be used to measure pain-related anxiety in pain patients and in healthy pain-free individuals in Libya. We hope that our study catalyses further research on this topic.

## Appendix 1

### The English version of PASS-20

PASS-20 Questionnaire Participant Code:

**Please rate the frequency of occurrence of each of the 20 behaviours listed below on a 6-point scale from 0 ‘never’ to 5 ‘always’**

## References

[1] Hasenbring MI, Verbunt JA (2010). Fear-avoidance and endurance-related responses to pain: new models of behavior and their consequences for clinical practice. Clin J Pain.

[2] Weisenberg M, Kreindler ML, Schachat R, Werboff J (1975). Pain: anxiety and attitudes in Black, White and Puerto Rican patients. Psychosom Med.

[3] Vlaeyen JW, Linton SJ (2000). Fear-avoidance and its consequences in chronic musculoskeletal pain: a state of the art. Pain.

[4] McCracken LM, Faber SD, Janeck AS (1998). Pain-related anxiety predicts non-specific physical complaints in persons with chronic pain. Behav Res Ther.

[5] Mccracken LM, Gross RT, Aikens J, Carnrike C (1996). The assessment of anxiety and fear in persons with chronic pain: a comparison of instruments. Behav Res Ther.

[6] McCracken LM, Zayfert C, Gross RT (1992). The pain anxiety symptoms scale: development and validation of a scale to measure fear of pain. Pain.

[7] Coons MJ, Hadjistavropoulos HD, Asmundson GJ (2004). Factor structure and psychometric properties of the pain anxiety symptoms scale-20 in a community physiotherapy clinic sample. Eur J Pain.

[8] Abrams MP, Carleton RN, Asmundson GJ (2007). An exploration of the psychometric properties of the PASS-20 with a nonclinical sample. J Pain.

[9] Cho S, Lee SM, McCracken LM, Moon DE, Heiby EM (2010). Psychometric properties of a Korean version of the pain anxiety symptoms scale-20 in chronic pain patients. International journal of behavioral medicine..

[10] Kreddig N, Rusu AC, Burkhardt K, Hasenbring MI (2015). The German PASS-20 in patients with low back pain: new aspects of convergent, divergent, and criterion-related validity. International journal of behavioral medicine.

[11] Wong WS, McCracken LM, Fielding R (2012). Factor structure and psychometric properties of the Chinese version of the 20-item pain anxiety symptoms scale (ChPASS-20). J Pain Symptom Manag.

[12] Abrams MP, Carleton RN, Asmundson GJ (2007). An exploration of the psychometric properties of the PASS-20 with a nonclinical sample. The journal of pain : official journal of the American Pain Society.

[13] Tashani OA, Alabas OA, Johnson MI (2010). Cold pressor pain responses in healthy Libyans: effect of sex/gender, anxiety, and body size. Gender medicine.

[14] Tabachnick BG, Fidell LS (2001). Using multivariate statistics.

[15] Bland JM, Altman DG (1996). Statistics notes: measurement error proportional to the mean. BMJ.

[16] Cronbach L (1984). Essentials of psychological testing.

[17] Costello A, Osborne J. Best practices in exploratory factor analysis: four recommendations for getting the most from your analysis. Pract Assess Res Eval 2005; 10. URL http://pareonline.net/getvn.asp. 2011;10(7).

[18] McCracken LM, Dhingra L. A short version of the Pain Anxiety Symptoms Scale (PASS-20): preliminary development and validity. Pain Research and Management. 2002;7(1):45–50. 10.1155/2002/517163 16231066

[19] Roelofs J, McCracken L, Peters ML, Crombez G, van Breukelen G, Vlaeyen JW (2004). Psychometric evaluation of the pain anxiety symptoms scale (PASS) in chronic pain patients. J Behav Med.

[20] Streiner DL, Norman GR, Cairney J. Health measurement scales: a practical guide to their development and use. 5th Edition. Oxford: Oxford University Press; 2014. p. 416.

[21] Keogh E, Barlow C, Mounce C, Bond FW (2006). Assessing the relationship between cold pressor pain responses and dimensions of the anxiety sensitivity profile in healthy men and women. Cogn Behav Ther.

[22] Goffaux P, Michaud K, Gaudreau J, Chalaye P, Rainville P, Marchand S (2011). Sex differences in perceived pain are affected by an anxious brain. Pain.

